# “Bionopoly” as a Gamechanger? Effects of Gamification on Learning Success, Motivation and Activation Among Medical Students in a Biochemistry Course

**DOI:** 10.1002/bmb.70045

**Published:** 2026-03-06

**Authors:** Eva Stapfer, Ernestine Saumweber, Achim Schneider, Susanne J. Kühl

**Affiliations:** ^1^ Institute of Biochemistry and Molecular Biology, Medical Faculty, Ulm University Ulm Germany; ^2^ Medical Faculty, Office of the Dean of Studies, Ulm University Ulm Germany

**Keywords:** ARCS‐model, biochemistry, experience sampling method (ESM), games, gamification, medical education, motivation

## Abstract

Gamification is characterized by the use of gaming elements in a non‐gaming context. This concept is commonly applied in teaching to create a more meaningful and activating learning environment. The major aim of this study was to compare possible effects of gamification on a traditional interactive teaching concept in a biochemistry course on students' learning. This study was performed in summer semester 2024 with fourth semester medical students at Ulm University in Germany. Students were divided into two homogenous study groups that differed in the teaching concept. In the control group, students received instruction through traditional, interactive face‐to‐face lessons that included open‐ended questions. In the gaming group, the quiz‐game “Bionopoly” was introduced and the students were taught using a gamified didactic concept. Learning success was measured by a multiple‐choice test at the beginning and the end of the course day. Satisfaction, motivation and a possible continuous activating effect were analyzed by an online evaluation and a handout questionnaire. Students of the gamified concept scored higher in the post‐knowledge test. Furthermore, the gamified concept motivated the student's significantly more when compared to the traditional teaching concept. Measured throughout the entire course day, gamification had a stabilizing effect on students' concentration, motivation and interest in biochemistry. All in all our study provides evidence that gamification is an attractive teaching concept for practical courses with an accompanying seminar.

## 1 Introduction

### 1.1 Games and Gamification

The popularity of gaming has grown in recent years. The game and puzzle industry in Germany demonstrated robust economic growth, with an increase of 9% in 2023 [1]. However, gaming is not merely a phenomenon that generates hype; it is also a useful tool for providing feedback, prompting action and evoking positive emotions [2]. Positive emotions such as amazement and curiosity, which are triggered during gameplay, support the motivation to learn something new [3]. This makes gaming an effective tool in education to encourage active learning [4].

The integration of gaming elements into a non‐gaming context, without the necessity of creating a game per se, is called gamification [4, 5]. In education, it represents a modern approach that integrates traditional didactical concepts with gaming elements. The specific combination of gaming elements is not limited and may include the use of gaming elements such as badges, (experience) points, quests, and more [4]. In contrast, a game that replaces the instruction entirely is defined as a serious game [5].

### 1.2 Gamification in Medical Education and Biochemistry

Somewhere between gamification and serious games, gamified classrooms have already been successfully used in medical training [4]. At the Medical Centre of the University Freiburg a game‐based e‐learning course was used to train medical students in urology. An accompanying study demonstrated that the implementation of gamification elements improved students' learning outcomes and promotes a positive attitude toward the course content. Furthermore, game‐based e‐learning facilitated effortless learning and enjoyment of a topic that might otherwise be perceived as unappealing [6]. At the Koyang University, a virtual reality program including defined rules and instructions has been established in which nursing students are trained in neonatal resuscitation. This gamified classroom encouraged the active participation of the learners, which increased their enthusiasm for the subject matter. Due to immediate correction, repetition and the experiences of success in this program, the student's self‐confidence was boosted [7]. When reviewing the literature on gamification in medical education, it is noticeable that most studies were conducted in a clinical setting and mainly cover topics in emergency medicine, surgery, and internal medicine [4, 6, 7].

Regardless of the central importance of clinical knowledge, preclinical topics are of fundamental importance for understanding medicine. Nevertheless, many students find them unattractive and insufficiently motivating. Thus, new and effective teaching concepts are particularly relevant in this area of medical teaching. Only in recent years more studies have been published concerning these preclinical topics, especially in biochemistry. Similar to studies in clinical teaching, for example, in urology [6] and emergency training [7], studies in medical biochemistry show, that educational games increase learning success, curiosity, collaboration, active engagement, and motivation of medical students [8, 9].

### 1.3 Biochemistry Practical Course at Ulm University

At the University of Ulm, the “practical course in biochemistry and molecular biology for students of human medicine” has been used a traditional interactive educational concept for some time. In the fourth preclinical semester of this degree program, medical students complete nine courses that are structured thematically. The topic of the course under investigation is “proteins and antibodies—precipitation, purification, electrophoresis, protein analysis and dot blot.” Every course day consists of three parts taking a total of 5.5 h: a pre‐seminar, a practical part in the laboratory, and a post‐seminar. The overall evaluation of the traditional concept by the students in the summer semester (SS) 2023 showed that the students find the topic “proteins and antibodies” hardly relevant for their medical studies and career. The students lost motivation and concentration during the 5.5 h and the teaching style in the seminars was described as passive and sometimes even demotivating. To tackle these comments in a modern, evidence‐based way, the traditional interactive concept was updated to a gamified version in the SS2024.

### 1.4 Aim of This Study

Therefore, the aim of this study was to investigate whether gamification improves the traditional teaching concept in the practical course day “proteins and antibodies” by comparing a gaming with a control group. In particular, we asked whether gamification can improve learning success, general satisfaction with the course and motivation of the medical students compared to the traditional teaching concept. We also tried to identify whether gamification can have a continuous activating effect during the 5.5 h on the medical students and thus increase their concentration, motivation and interest over a course day.

## 2 Materials and Methods

### 2.1 Course Description

The didactic concept of the “biochemistry and molecular biology practical course for students of human medicine” is designed to offer medical students the opportunity to combine the theoretical input of seminars directly with the experience of a laboratory course. In the fourth preclinical semester of the degree program, medical students complete nine course days that are structured thematically and taught in student groups of approximately 20 participants. Each of the nine course days lasts about 5.5 h. The course day 9 has the topic “proteins and antibodies—precipitation, purification, electrophoresis, protein analysis and dot blot”.

One course day is divided into three parts:

1. *Pre‐seminar:* Theoretical part giving the biochemical background for the coming experiments.
2. *Laboratory:* Students perform experiments under the guidance of tutors. This is not part of the present study.
3. *Post‐seminar:* To reflect on and discuss the experimental results and to clarify questions.

The two lecturers—Prof. Dr. Susanne Kühl (S.J.K.) and Dr. Ernestine Saumweber (E.S.)—used a standardized slide presentation for pre‐ and post‐seminars to ensure the same content for all student groups.

### 2.2 Study Design Including Test Tools

This study compares a traditional interactive classroom with a gamified classroom in SS2024 from April to June. The medical students were randomly and equally divided into two study groups, the control group (CG, five student groups with *N* = 74 medical students) and the gaming group (GG, five student groups with *N* = 60 medical students).

**FIGURE 1:**
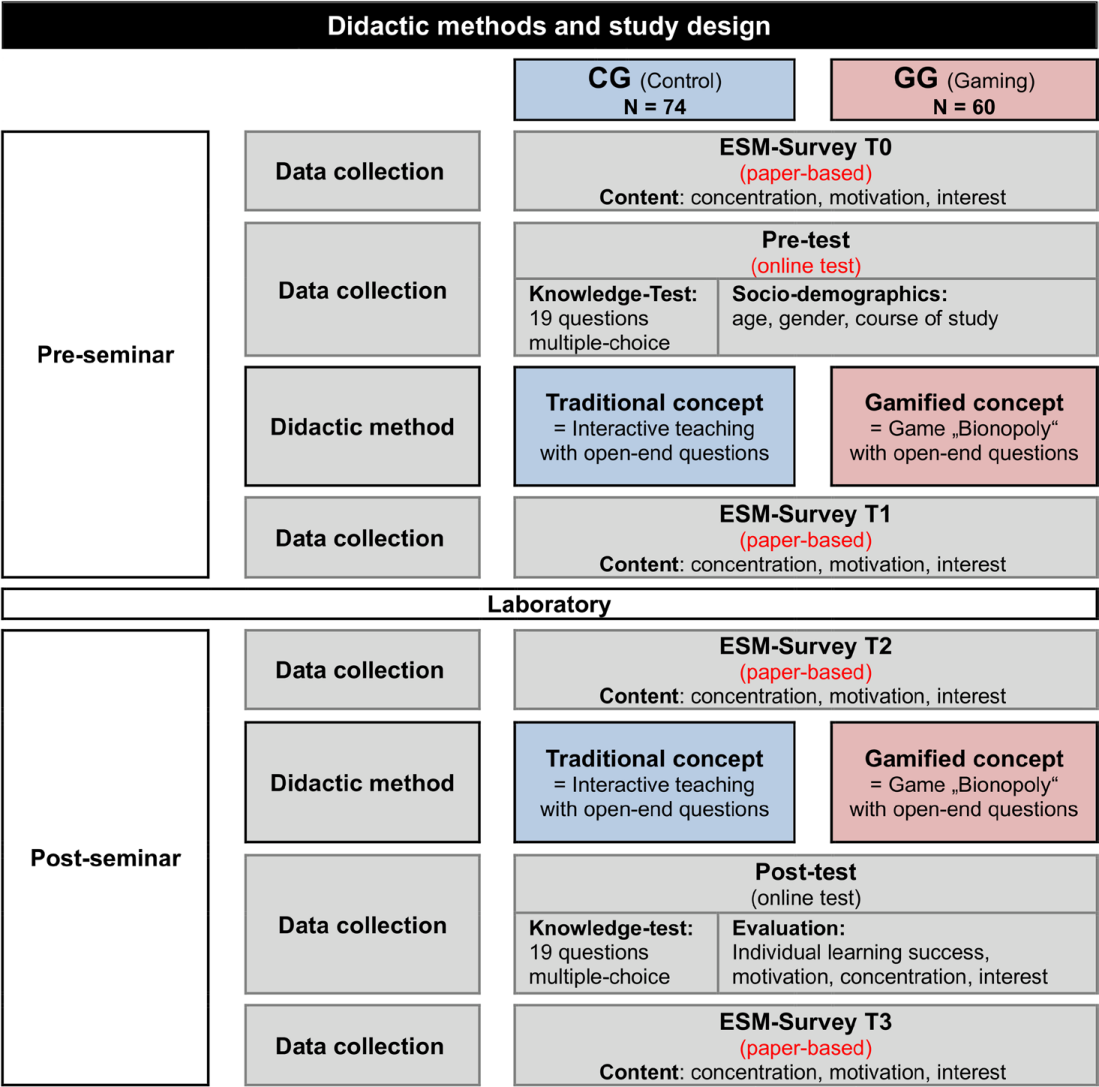
Didactic methods and study design. This study was performed with medical students in the summer semester of 2024, from 17 April to 14 June. In contrast to the traditional approach, the open‐end questions were asked as part of the game “Bionopoly.” In addition, the students in the gaming group were divided into teams of four to five students of the gamified lessons, with points being given for answering the questions correctly. There were five possible answers to the multiple‐choice questions, which were categorized as either type A (positive) or type B (negative). CG, control group; ESM, experience sampling method; GG, gaming group; *N*, number of students; t0, time 0; t1, time 1; t2, time 2; t3, time 3.

For analyzing the teaching concepts, three tools were used: a multiple‐choice online test on knowledge acquisition, an online questionnaire to collect data on demographics, motivation, factors on influencing learning success and satisfaction, and a paper‐based ESM‐survey (experience sampling method) to record the status of concentration, motivation and interest at four different points in time along the pre‐ and post‐seminars. All online tests were completed on the platform “unipark” (EFS Survey Software, version 21.2, Tivian XI GmbH, Cologne, Germany). We granted the students access via QR codes. The paper‐based ESM‐Survey was distributed as a handout at the beginning of the pre‐seminar. Identical tests were used for both study groups (see Figure 1).

### 2.3 Control Group: Traditional Didactic Concept

The control group consists of 74 participants (assigned to five student groups). S.J.K. lectured two and ES three control student groups. The traditional didactic concept is implemented as interactive frontal teaching in which students are encouraged to answer 36 open‐end questions relating to the topics of amino acids, proteins and enzymes. The standardized slide‐presentation was designed neutrally for this study group. A slide with an open‐end question was always followed by a slide with the correct answer including an explanation.

### 2.4 Gaming Group: Gamified Didactic Concept

For the gamified didactic concept, the game named “Bionopoly” was developed. The gaming group consists of 60 medical students (assigned to five student groups). Two student groups were taught by S.J.K. and three student groups by E.S. In the gaming group game elements (colors, street names, images) were added to the standardized slide‐presentation with the same 36 open‐end questions as in the control group. As part of the game “Bionopoly” each student group was subdivided into 4–5 teams of 4 to 5 students.

### 2.5 Gaming Group: „Bionopoly “Concept

**FIGURE 2:**
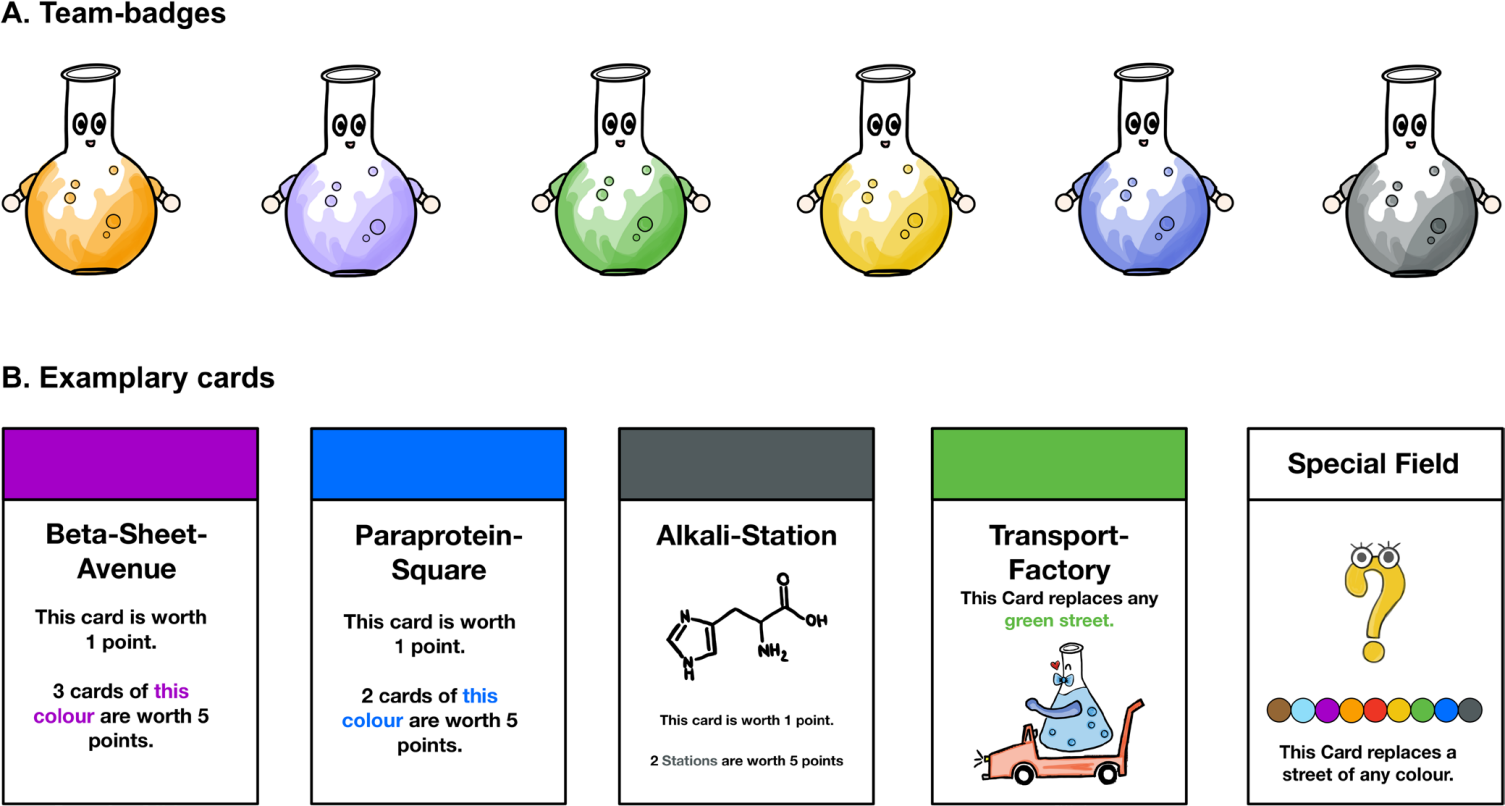
Team badges and exemplary cards of the game “Bionopoly.” The course was divided into four to five teams, each consisting of four to five students. (A) The badges that the teams received. The “Bionopoly” game board contains different kinds of squares: 22 streets, four railway stations, seven factories and three special fields. Each square represents a quiz‐question. (B) Examplary cards for every kind of squares that were given to students if they answered the corresponding quiz‐question correctly. The design of the game is based on the game Monopoly, which was originally conceived by Elisabeth Maggie Phillips.

**FIGURE 3:**
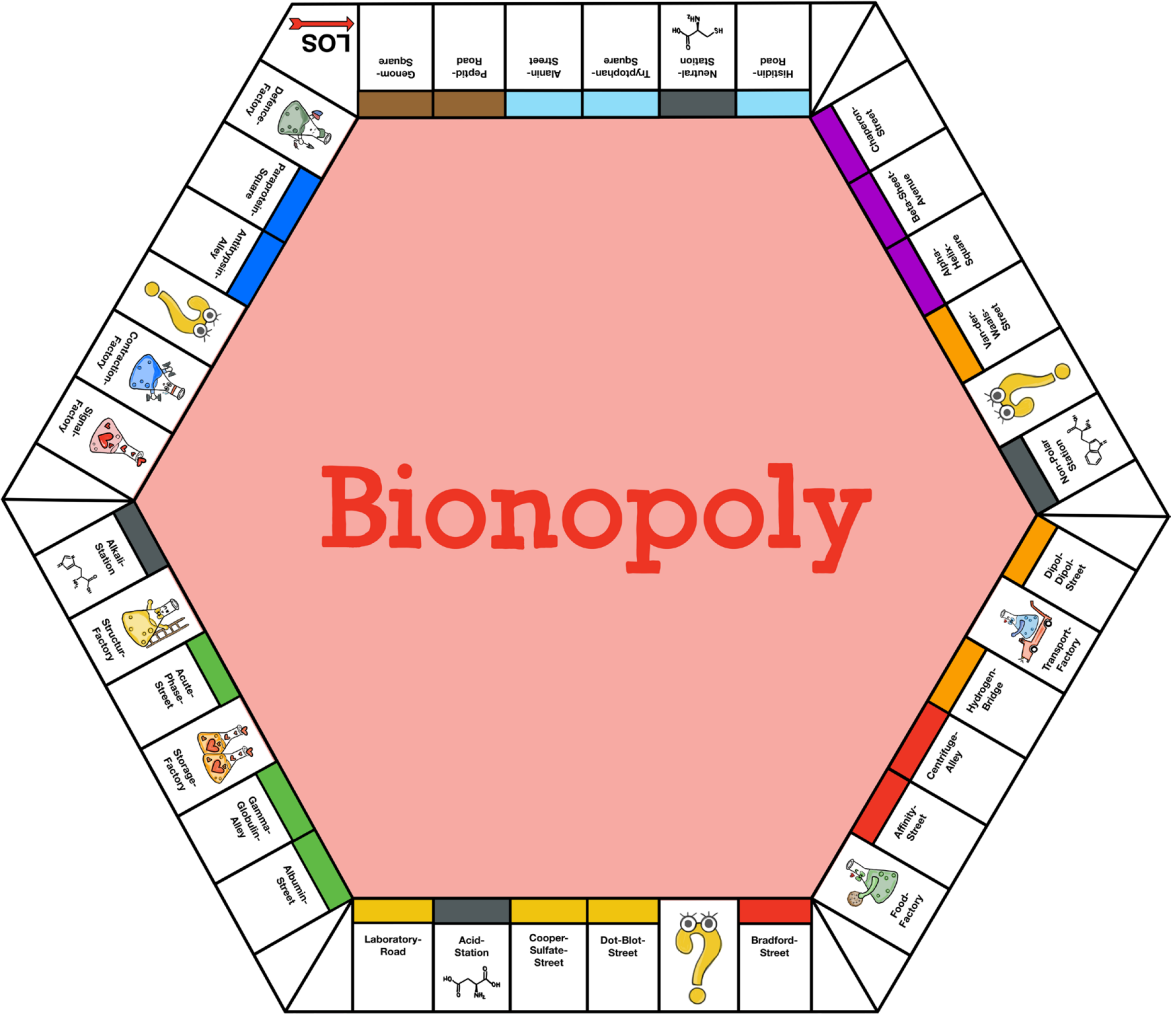
Game board “Bionopoly.” The game “Bionopoly” contains 36 open‐ended questions, each represented as a single square on the game board. The game continues clockwise from the starting point in the top left corner. The design of the game is based on the well‐known game Monopoly, which was originally conceived by Elisabeth Maggie Phillips.

1. *Design:* Based on the board game Monopoly, the adaptation used in this study—“Bionopoly”—was integrated as a quiz game. It comprises a total of 36 questions relating to the topics of amino acids, proteins, and enzymes (same questions as for the control group). Compared to the original, the game was adapted in terms of names, graphics, and content. The student group of one course day was divided into 4–5 teams, each consisting of 4–5 students. Every team got badges that were represented by conical flasks in different colors (Figure 2A). The game board contains groups of colored squares and single special squares (Figure 3). The special squares include four railway stations, seven factories, and three special fields. Each square represents a quiz question. There was a corresponding card for each square (Figure 2B), which was given to students if they answered the quiz question correctly.
2. *Rules:* Each team consists of a maximum of five medical students. The squares on the game board are played one after the other, starting at the field “LOS.” Each square corresponds to one open‐end question. The fastest student (indicated by raising the hand) has the opportunity to answer the question on behalf of their team; if the answer is correct, the group receives the card that corresponds to the square on the board. If the answer is incorrect, the second fastest student has the opportunity to answer.
3. *Scoring:* Each square/card scores one point. If the students collect all cards of the same color the row scores five points. Factories and special fields can be used as jokers for other colors. Factories indicate which color they can replace. Special fields can be used as jokers for any color of the game. Two railway stations scores five points.
4. *Implementation:* The game was played in the pre‐ and post‐seminars, supported by the game board (on the blackboard, Figure 3) and presentation slides containing the 36 quiz‐questions. A slide with an open‐end question was always followed by a slide with the correct answer. At the beginning of the semester, both lecturers performed one practice session with a group of students in April 2024 to familiarize themselves with the game and to optimize the process. The practice sessions' data was not included in the study data.

### 2.6 Data Collection

Students were asked to participate voluntarily in the evaluation. For all evaluation questions, it was taken ensured that concrete reference was made to the teaching concept used in the pre‐ and post‐seminar. The main components of the evaluation are described in more detail below:

### 2.7 Socio‐Demographic Data

The students were asked about their age, gender and course of study at the end of the pre‐knowledge‐test. This data was used to determine whether the study groups were comparable.

#### 2.7.1 Analysis of the Learning Success and Corresponding Impact Factors

To analyze the objective knowledge acquisition, a knowledge test with 19 knowledge questions based on the topic of the practical course day was created. The test was performed online before (pre‐test) and after the course (post‐test) with a time limit of 15 min. The questions were presented in a multiple‐choice format. After both tests the students received immediate feedback in form of an overall score.

To analyze the subjective learning success, the students answered a question about impact factors on learning success. Four factors (interactive teaching, group dynamics, presentation slides, and teacher's instructional style) were rated by the students (1 = negative impact, 2 = no impact, 3 = positive impact).

#### 2.7.2 Analysis of the General Satisfaction

Students' reported their general satisfaction by rating the course as a whole from 1 (very good) to 6 (poor) in the online post‐test. A free text option allowed students to give positive and negative feedback on the teaching concept.

#### 2.7.3 Analysis of the Motivation

To analyze the medical students' general motivation and factors, that motivate them to stay focused, several items in the online post‐test were integrated. The questions on general motivation were based on Keller's ARCS model. It describes motivation as a concept with the four dimensions Attention, Relevance, Confidence and Satisfaction and provides an instructional design for improving lectures [10]. The model has been successfully used in many studies concerning education and gamification [3, 4, 7, 8] and offers the possibility to analyze learners' motivation in detail and to develop targeted improvement strategies. For every dimension, the evaluation sheet included one question, in total four questions. Every dimension was rated on a six‐point Likert‐type scale from 1 (strongly disagree) to 6 (strongly agree).

One additional item—unrelated to the ARCS model—asked about the motivational factors. Students were able to select from four aspects: fun, group dynamics, intrinsic motivation and competition. The opportunity was given to select multiple factors from the options listed.

#### 2.7.4 Analysis of a Possible Continuous Activating Effect With the Experiential Sampling Method

To detect a possible effect of gamification on the students' active participation throughout the course, their self‐assessment regarding concentration, motivation and interest continuously throughout the day was measured. This type of questionnaire in general is based on the experience sampling method (ESM) developed by Suzanne Prescott in 1977. It describes questionnaires that ask for participants subjective perception: especially for their thoughts, feelings, behaviors continuously over a short period of time. A specific design of the questionnaire is not mandatory [11]. In this study, a paper‐sheet questionnaire with three items (e.g., “My concentration is high”) was answered by the students on a six‐point Likert‐type scale (1 = strongly disagree, 6 = strongly agree) at four fixed points over time: before and after the pre‐seminar and before and after the post‐ seminar (see Figure 1).

The questionnaires can be found in Supporting Information A.

### 2.8 Data Analysis and Statistics

Data from individual participants with multiple missing responses were excluded. Missing information on age alone was considered to be a minor issue and their data was included in the study. The final study compares the results of the control group of 74 students with the gaming group of 60 students.

Due to the non‐normal distribution of the knowledge test data for both groups, as indicated by the Kolmogorov–Smirnov test (*p* < 0.05), the non‐parametric Wilcoxon signed‐rank test was used to compare related samples. To compare independent samples, the Mann–Whitney *U* test was applied. We used the difference between pre‐ and post‐test scores as a measurement for knowledge acquisition. The general motivation of a student was determined by calculation the mean of the answers to the four questions on the dimensions of motivation (according to Keller). The general motivation was then compared between the two study groups using the Mann–Whitney *U* test. Additionally, the students' responses in the free‐text fields were categorized and quantified, based on themes of praise, criticism, and content. A *p*‐value of *p* < 0.05 was considered to be significant. Data was analyzed by using the IBM SPSS Statistics Version 29 for Windows.

### 2.9 Ethics and Consent

The ethics committee of Ulm University confirmed that an ethics approval was not necessary for this study. The data collection was voluntary and anonymous, and the students were not paid for their participation. Before participating in the knowledge test (pre‐test), the students were asked to give their consent to process the data. Participation and consent could be withdrawn at any time.

## 3 Results

Based on the sociodemographic data (age, gender, course of study), the two study groups can be considered comparable (Table 1).

**TABLE 1:** Demographic data of the final data set.

	Control group	Gaming group	Comparison
Number of students (*N*)	74	60	—
Age (years)	21.76 ± 3.05	21.26 ± 2.08	n.s., *p* = 0.413
Men	59.5%	61.7%	n.s., *p* = 0.500
Women	40.5%	36.7%	
Not specified	—	1.7%	
Course of study: Medicine	100%	100%	Constant

*Note:* The data originates from the pre‐test. All data with complete questionnaires and questionnaires with only missing the age of the participant were included. The study was performed in the summer semester, from 17 April to 14 June 2024. **Blue:** The students in the control group were educated using an interactive, open‐end question format. **Red:** The students in the gaming group were taught using the “Bionopoly” game with open‐end questions. The data are presented as mean values with standard deviations or percentages. The Kolmogorov–Smirnov test was used for statistical analysis.

Abbreviations: *N*, number of students; n.s., not significant; *p*, statistical significance.

### 3.1 Analysis of the Learning Success

**FIGURE 4:**
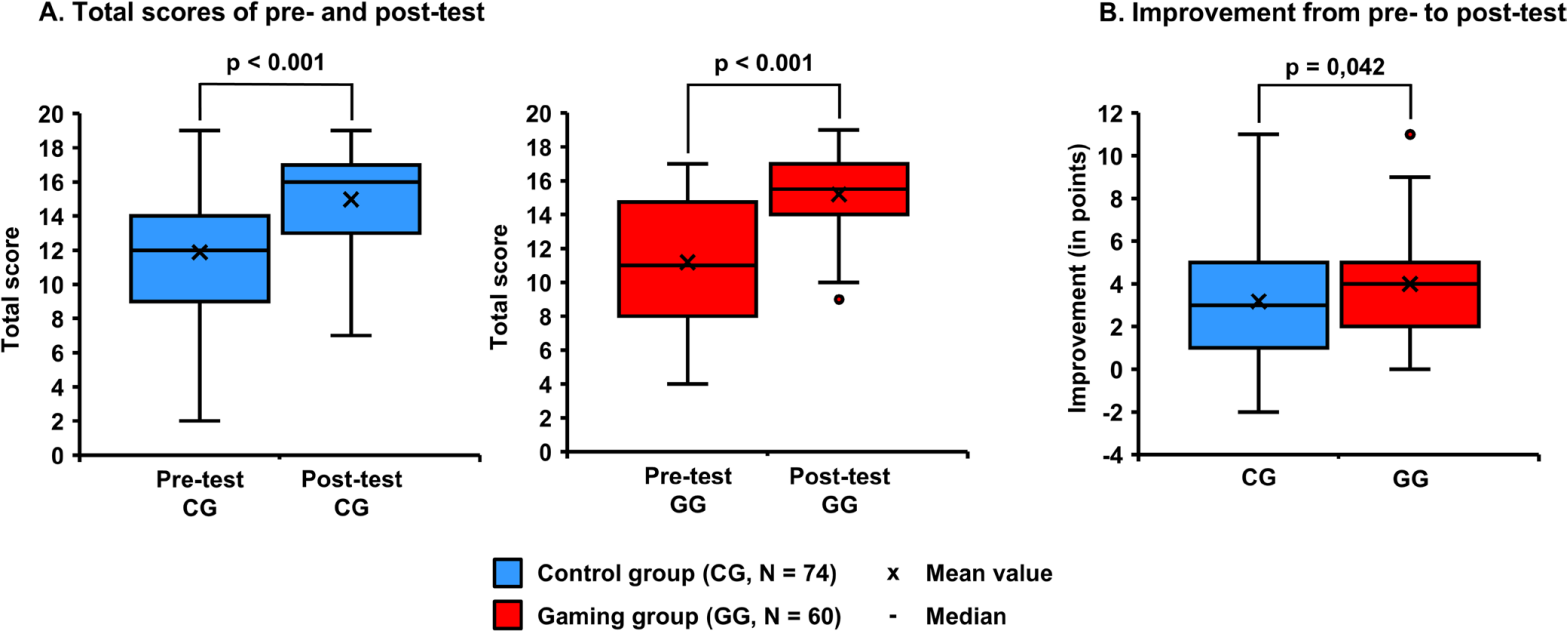
Results of pre‐ and post‐test. The knowledge test consisted of 19 questions presented in a multiple‐choice format. Only one answer was correct, following type A (positive) or type B (negative). (A) Both the control group (blue) and the gaming group (red) demonstrated a statistically significant increase in the total scores between the pre‐ and post‐test. The *Y*‐axis depicts the total score of the knowledge test. The mean values are indicated by *X* in the graphs, while the medians are represented by a solid line. Error bars are included to represent the standard deviation in each case. The Wilcoxon signed‐rank test was used for the purpose of statistical analysis. (B) The data shows a statistical difference between the improvement of the two group from pre‐ to post‐test. The Mann–Whitney *U* test was used for statistical analysis. *p*, statistical significance; CG, control group; GG, gaming group; *N*, number of students.

In order to evaluate the learning success of the two study groups, the results of the knowledge tests were analyzed (see Figure 4). The control group showed a significant improvement (*p* < 0.001) from the pre‐ (M_Pre_ = 11.88 ± 4.03) to the post‐test (M_Post_ = 14.97 ± 3.20) as well as the gaming group (M_Pre_ = 11.20, M_Post_ = 15.20, *p* < 0.001) (Figure 4A). The control group improved on average by three points and the gaming group by four points. With a *p*‐value of 0.042, there is a significant difference between the control and gaming group in regard to the difference in the mean scores from pre‐ to post‐test showing that students in the gamified seminar achieve higher learning success as students of the control group (Figure 4B).

**FIGURE 5:**
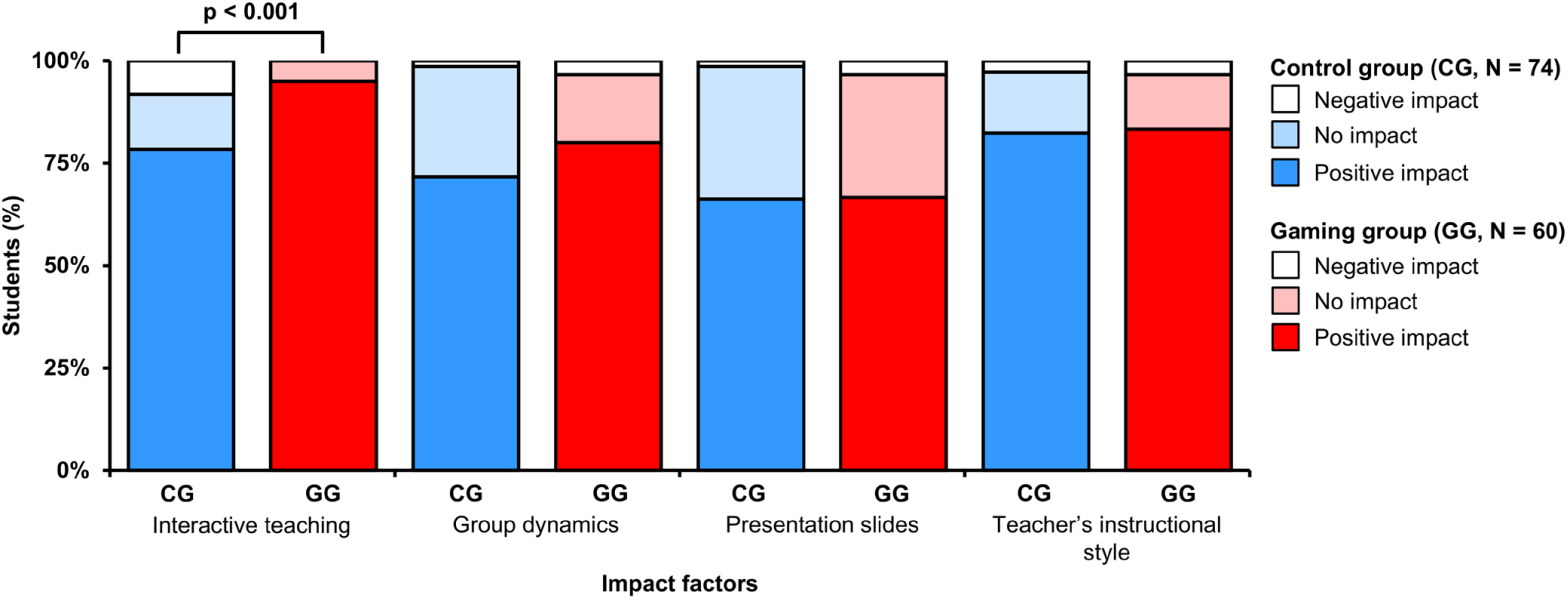
Impact factors on learning success. The impact factors (*X*‐axis) could be evaluated either as negative, no, or positive in terms of their impact. The *Y*‐axis shows the number of students as a percentage. The Mann–Whitney *U* test was used for statistical analysis. *p*, statistical significance; CG, control group; GG, gaming group; *N*, number of students.

To distinguish from other studies, we tried to identify factors that influence learning success in both groups. Students in the gamified concept rated the factor interactive teaching as a positive influence significantly more often (*p* = 0.005) than the control group (see Figure 5). Moreover, 90% of the students in the gamified seminars consider the game “Bionopoly” as a positive impact on their learning success (data not shown).

### 3.2 Analysis of the General Satisfaction

The overall evaluation of the course was based on a scale of 1 (very good) to 6 (unsatisfactory). The mean rating of the control group was 2.5 (M = 2.61 ± 1.10), while the mean rating of the gaming group was 2.00 (M = 2.05 ± 0.85). The results showed a statistically significant difference in favor of the gamified concept (*p* = 0.003, data not shown).

Writing a free text was used by 32.4% (24 of *N* = 74) of the students of control group and 15.0% (9 of *N* = 60) of the students in the gaming group. Overall, four main points of criticisms were identified: duration, structure of the course, repetition of content and loss of concentration. In both groups the long duration of the course was criticized the most. In general, there was more criticism than praise from the students. The control group praised the course for being interesting and mentioned the particular reasons such as the slides or the lecturer. In comparison, the gaming group complimented the course as “didactically great,” for example, and described the course and the game “Bionopoly” in particular as “[…] a lot of fun” (data shown in Supporting Information B).

### 3.3 Analysis of Students´ Motivation

**FIGURE 6:**
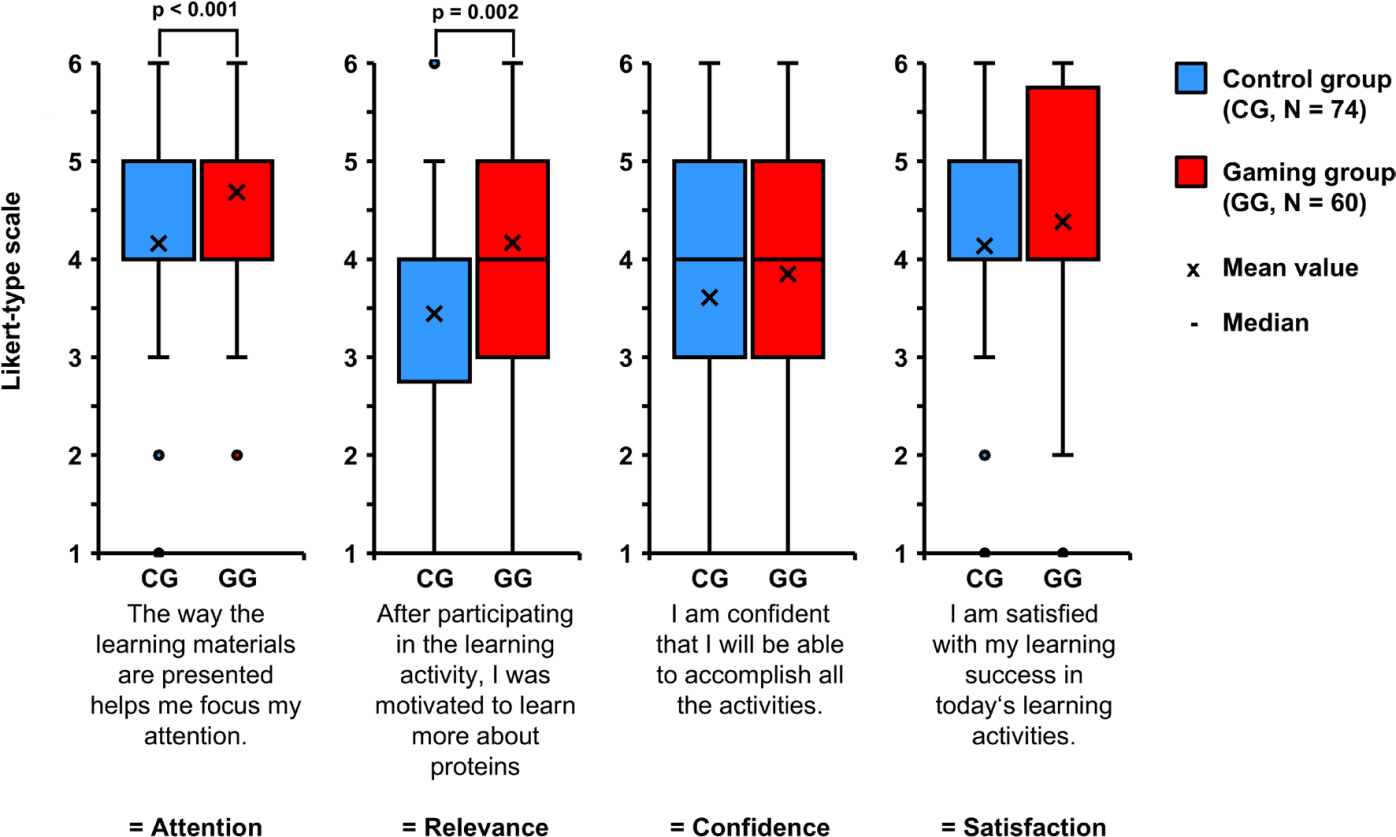
Evaluation of motivation in the context of the four dimensions of Keller's ARCS model: Attention, Relevance, Confidence and Satisfaction. The four dimensions (*X*‐axis) were rated by the students on a Likert scale from 1 (strongly disagree) to 6 (strongly agree). The mean values are indicated by *X*s on the graphs, while the medians are represented by solid lines. Error bars illustrate the standard deviation. The Mann–Whitney *U* test was employed for statistical analysis. *p*, statistical significance; CG, control group; GG, gaming group; *N*, number of students.

Students’ motivation was measured using Keller's four dimensions such as Attention, Relevance, Confidence and Satisfaction. General motivation of the control and gaming group was tested by comparing the mean of all four dimensions of both groups. The gaming group (M = 4.28 ± 0.94) showed significantly higher general motivation than the control group (M = 3.83 ± 1.01, *p* = 0.026, data not shown). When the components of Keller's model were analyzed separately, a significant difference was found between the two study groups in the dimensions Attention (M_CG_ = 4.16 ± 1.11, M_GG_ = 4.75 ± 0.91, *p* = 0.001) and Relevance (M_CG_ = 3.45 ± 1,28, M_GG_ = 4.20 ± 1.31, *p* = 0.002) (Figure 6). To sum up, in the student's opinion the gamified concept attracts attention and emphasizes the relevance of the topic more than the traditional concept.

**FIGURE 7:**
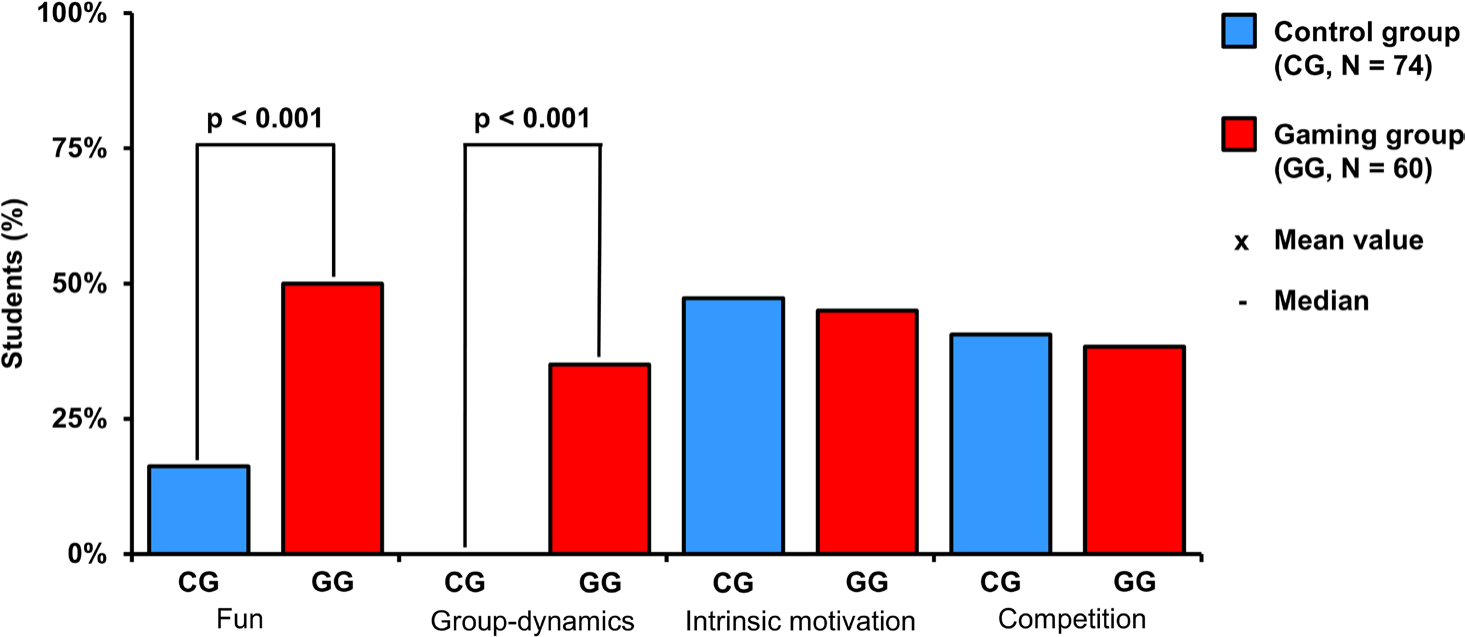
Analysis of motivational factors. The *Y*‐axis shows the percentage of students who indicated that the listed factors (*X*‐axis) were motivating. The Mann–Whitney *U* test was performed for statistical evaluation. *p*, statistical significance; CG, control group; GG, gaming group; *N*, number of students.

Next, we asked the students which factors—fun, group dynamics, intrinsic motivation, competition—they found motivating and observed that the gaming group reported the factors fun (GG = 50%, CG = 16.2%, *p* < 0.001) and group dynamics (GG = 38.3%, CG = 0%, *p* < 0.001) significantly more often than the control group (see Figure 7).

### 3.4 Analysis of a Possible Continuous Activating Effect of Gamification

**FIGURE 8:**
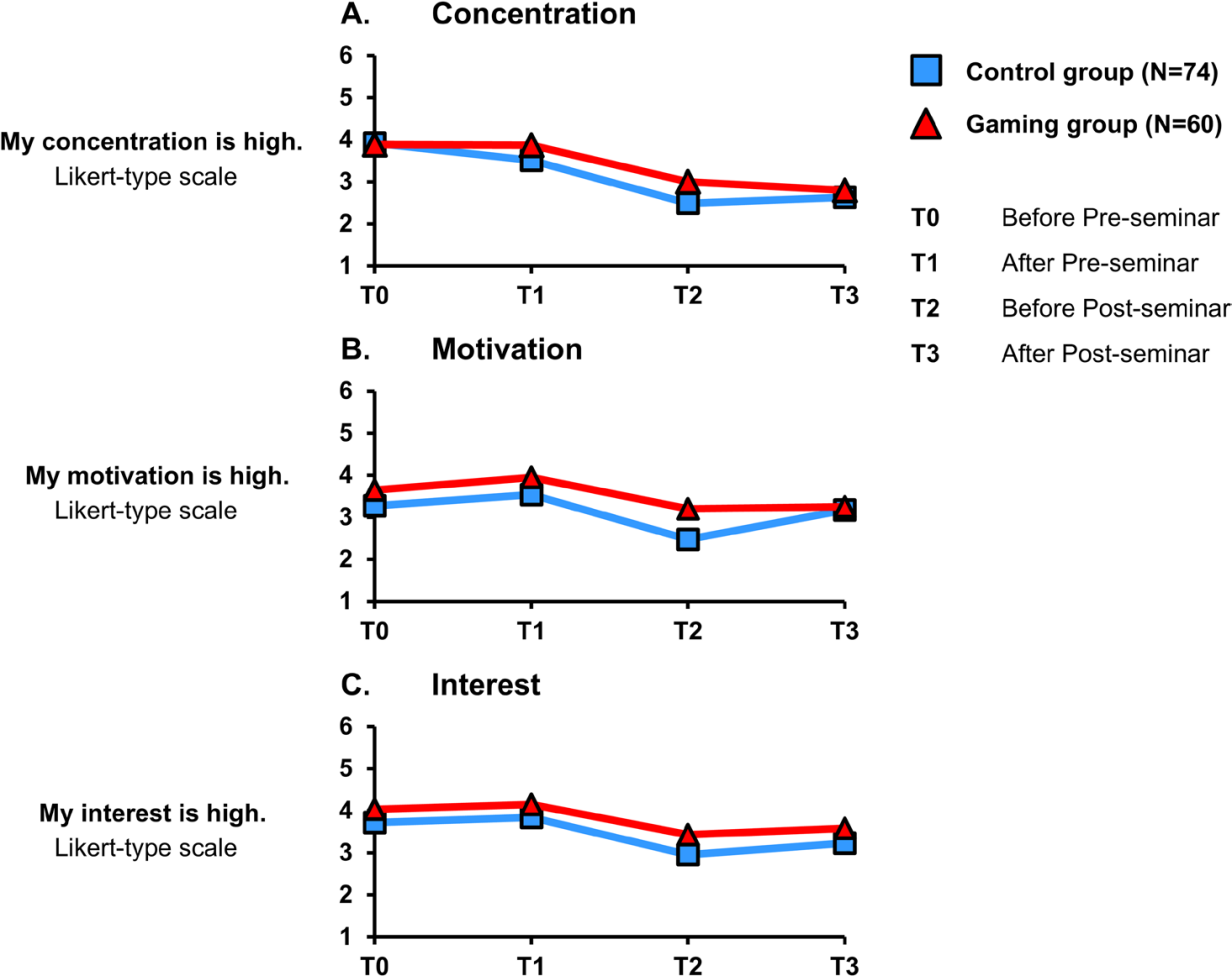
Concentration, motivation and interest throughout the course. The three items A–C (Y‐axis) were rated by the students on a Likert‐type scale from 1 (strongly disagree) to 6 (strongly agree). The ESM‐Survey (experiential sampling method) was conducted at four different times (T0–T3), both before and after the pre‐seminar and the post‐seminar. The laboratory practical course was performed between the second and third surveys. The blue lines and squares illustrate the longitudinal progress of the control group. The red lines and triangles illustrate the longitudinal progress of the gaming group. *N* number of students.

The active participation of the students was analyzed during the course by observing their concentration, motivation and interest (Figure 8). For this purpose, an ESM‐survey was used at four specific points in times: T0 (before the pre‐seminar), T1 (after the pre‐seminar), T2 (before the evening seminar) and T3 (after the evening seminar). The descriptive analysis reveals that all three evaluated variables show similar trends in both groups with a less pronounced decline in the gaming group. Concentration decreased over time in both groups: in the control group (T0 = 3.92 ± 1.04 to T3 = 2.64 ± 1.25) and in the gaming group (T0 = 3.88 ± 1.18 to T3 = 2.80 ± 1.52). Motivation also diminished over time in the control (T0 = 3.27 ± 1.05 to T3 = 3.18 ± 1.52) and in the gaming group (T0 = 3.65 ± 1.18 to T3 = 3.25 ± 1.54). There was also a decline in interest in both groups: in the control group (T0 = 3.72 ± 1.04 to T3 = 3.23 ± 1.34) and in the gaming group (T0 = 4.03 ± 1.06 to T3 = 3.58 ± 1.41).

## 4 Discussion and Limitations

This study shows that the gaming group benefits from the teaching concept in terms of knowledge acquisition. In addition, students' general satisfaction in the gamified concept was higher than in the traditional concept. Students state that gamification increases their motivation (according to Keller's model), especially with regard to their attention and perception of the topic's relevance. Furthermore, gamification seems to have a slight stabilizing effect on students' concentration, motivation and interest in biochemistry. All in all, this study demonstrates that gamification can be motivating for students of human medicine in a seminar that frames a practical course in biochemistry and molecular biology.

### 4.1 Comparison of Learning Success

The existing literature supports the hypothesis that the use of gamification can lead to improved knowledge acquisition in biochemistry. Games such as “Livogena” and “CARBGAME” at Panimalar Medical College improved students’ performance significantly [8, 9]. Similarly, “Histopoly” at the University of Porto showed short‐term increase in knowledge by the serious game [12]. In our study, the gaming group also showed a significantly higher learning success than the control group. However, the greater increase in knowledge could be partly due to the fact that the gaming group started with lower scores in the pre‐test.

Students described that former games like “Catalyze!” and “CARBGAME” promote engagement and collaboration, although they have not always been compared to control groups [8, 9]. In our study, students in the gaming group perceived “interactive teaching” more positively than those in the control group, suggesting that this factor contributes to subjective learning success. Future research should further investigate which elements of gamification contribute to learning success.

### 4.2 Comparison of General Satisfaction

This study supports existing evidence that gamified teaching increases student satisfaction in biochemistry education. In games such as “Livogena,” “CARBGAME,” and “Histopoly” students have shown high levels of satisfaction and better internal evaluations compared to non‐gamified courses [8, 9, 12]. Similarly, the gaming group in our study gave more positive feedback and enjoyed the course more than the control group, although both groups rated the course positively overall. Notably, both groups criticized the length and structure of the course, but the control group criticized it more often. Due to the overall imbalance in the numbers of comments between the two groups, further research is required to evaluate whether gamification can improve lower rated courses and be successfully applied to other biochemistry topics.

### 4.3 Comparison of Students´ Motivation

Many studies have confirmed that gamification significantly increases students’ motivation, with games such as “Livogena” and “CARBGAME” achieving motivation in over 96% of the students [8, 9], but lacking a control group. However, few studies have compared motivation between gamified and traditional teaching concepts; for example, the “Histopoly” study by Marcos et al. shows higher motivation in the gamified group than in a control group [12]. The present study complements these findings by showing that the gamified concept using “Bionopoly” significantly increases students’ motivation compared to a traditional concept. Using Keller's ARCS model, it was found that “Attention” and “Relevance” were particularly positively affected by the game. Although only one question per ARCS dimension was used in the study—in contrast to the full Instructional Materials Motivation Survey (IMMS) tool used in other studies—it still supports the conclusion that gamification can effectively capture students’ attention and emphasize the relevance of the topic. More robust studies using the full ARCS framework are needed to validate these findings, particularly in biochemistry courses for medical students.

While other studies only suggest a connection between fun, the students in our study specifically named “fun” and “group dynamics” as motivating factors.

### 4.4 Continuous Activating Effect of Gamification

In the past, medical students at the University of Ulm criticized a course day for being too long. This study uniquely focused on tracking concentration, motivation and interest throughout one course day. Although these factors declined in both groups, students in the gamified group continuously reported higher levels across all factors. The most remarkable drop occurred during the practical part in the laboratory when no gamified elements were used. While gamification did not resolve the issue of perceived course length, it proved slightly more effective than the traditional teaching concept in sustaining student engagement. Future research should investigate alternative course structures or gamified concepts that could alleviate the sense of time burden. In particular, our course day could benefit from testing whether integrating gamified exercises during periods of waiting—such as while laboratory results are processed‐ might positively influence student's perception of time.

### 4.5 Bionopoly as Gamified Didactic Concept

Monopoly, a widely known and easily adaptable board game, has been successfully used in various fields of medical education such as physiology, anatomy and histology [12, 13, 14]. However, to our knowledge, this study is the first to use a modified version of Monopoly, “Bionopoly,” for biochemistry education. Our students quickly understood the game mechanics, allowing them to focus on the content of the topic biochemistry. The Monopoly format proved to be more than just visually appealing—it supported the students’ learning process.

## 5 Conclusion and Outlook

This study contributes to the growing body of evidence on the effectiveness of gamification in enhancing learning success, overall students’ satisfaction, and motivation. We identified new potential impact factors on learning, defined the most affected motivational dimensions (according to Keller [10]) and outlined specific motivational factors in a gamified concept. Overall, our findings suggest that gamified teaching is particularly effective for enhancing engagement in courses perceived as dry or less stimulating. However, the concept of gamification and the mechanisms through which it operates remain only partially understood. It is essential to uncover how gamification exerts its positive effects and how these are processed both cognitively and physiologically. Effective implementation requires careful selection between gamified elements and intended learning success. It is equally important not to underestimate the complexity and effort involved in implementing a new teaching concept.

## Funding

The authors have nothing to report.

## Conflicts of Interest

The authors declare no conflicts of interest.

## Supporting information


**Supporting Information: A.** Evaluation sheets.
Questionnaire 1: ESM‐Survey: (paper‐based). Questionnaire 2: Pre‐test: (online). Questionnaire 3: Post‐test: (online).

**Supporting Information: B.** Comments—General satisfaction.
In the control group, 24 of the participants (N = 74) used the comment function, which corresponds to 32%, compared to nine participants (N = 60) in the gaming group who commented, which corresponds to 15%. The texts were sorted according to the content of the improvement suggestion and according to praise. A text can be categorized both as a suggestion for improvement and as praise. If a comment addressed several of the topics, the comment was assigned to all of these topics and categorized under NK (for the number of comments on this topic). The number N in the table stands for the total number of comments made in this group. According to the system above, N does not correspond to the sum of NK.

## Data Availability

The data that support the findings of this study are available from the corresponding author upon reasonable request.

## References

[1] Spiele‐Markt Kehrt Zurück Auf Wachtumspfad. In: Spieleverlage EV, 2024, https://www.spieleverlage.com/spiele‐markt‐kehrt‐zurueck‐auf‐wachtumspfad/.

[2] R. Koster and W. V. Wright , A Theory of Fun for Game Design, 2004 (O'Reilly and Associates, 2013).

[3] U. Durrani , O. Hujran , and A. S. Al‐Adwan , “CrossQuestion Game: A Group‐Based Assessment for Gamified Flipped Classroom Experience Using the ARCS Model,” Contemporary Educational Technology 14 (2022): 8–10.

[4] A. E. J. van Gaalen , J. Brouwer , J. Schönrock‐Adema , T. Bouwkamp‐Timmer , A. D. C. Jaarsma , and J. R. Georgiadis , “Gamification of Health Professions Education: A Systematic Review,” Advances in Health Sciences Education 26 (2021): 683–711, 10.1007/s10459-020-10000-3. 33128662 PMC8041684

[5] S. Deterding , R. Khaled , L. Nacke , and D. Dixon , Gamification: Toward a Definition, 2011, pp. 12–15.

[6] M. Boeker , P. Andel , W. Vach , and A. Frankenschmidt , “Game‐Based E‐Learning Is More Effective Than a Conventional Instructional Method: A Randomized Controlled Trial With Third‐Year Medical Students,” PLoS One 8 (2013): e82328, 10.1371/journal.pone.0082328. 24349257 PMC3857775

[7] S.‐Y. Yang and Y.‐H. Oh , “The Effects of Neonatal Resuscitation Gamification Program Using Immersive Virtual Reality: A Quasi‐Experimental Study,” Nurse Education Today 117 (2022): 105464, 10.1016/j.nedt.2022.105464. 35914345 PMC9259066

[8] K. M. Surapaneni , ““CARBGAME” (CARd & Board GAmes in Medical Education) as an Innovative Gamification Tool for Learning Clinical Enzymology in Biochemistry for First Year Medical Students,” Biochemistry and Molecular Biology Education 52 (2024): 666–675, 10.1002/bmb.21857. 39136227

[9] K. M. Surapaneni , “Livogena: The Ikteros Curse—A Jaundice Narrative Card and Board Game for Medical Students,” MedEdPORTAL : Publications of the Association of American Medical Colleges 20 (2024): 11381, 10.15766/mep_2374-8265.11381. PMC10844581 38322827

[10] J. M. Keller , “Development and Use of the ARCS Model of Instructional Design,” Journal of Instructional Development 10 (1987): 2–10, 10.1007/BF02905780.

[11] M. Csikszentmihalyi , R. Larson , and S. Prescott , “The Ecology of Adolescent Activity and Experience,” Journal of Youth and Adolescence 6 (1977): 281–294, 10.1007/BF02138940. 24408457

[12] R. Marcos , A. Gomes , M. Santos , and A. Coelho , “Histopoly: A Serious Game for Teaching Histology to 1st Year Veterinary Students,” Anatomical Sciences Education 18 (2024): 229–240, 10.1002/ase.2545. 39782021

[13] E. G. Anyanwu , “Anatomy Adventure: A Board Game for Enhancing Understanding of Anatomy,” Anatomical Sciences Education 7 (2014): 153–160, 10.1002/ase.1389. 23878076

[14] M. Dong , J. Uricheck , and U. Vaid , “PULMONOPOLY: A Game‐Based Approach to Teach and Reinforce Basic Concepts in Pulmonary Medicine to Medical Students,” Chest 164 (2023): A3847, 10.1016/j.chest.2023.07.2507. PMC11842520 39991754

