# Supplementary material for: “Bionopoly” as a Gamechanger? Effects of Gamification on Learning Success, Motivation and Activation Among Medical Students in a Biochemistry Course
Source: Biochem Mol Biol Educ. 2026 Mar 6;54(3):243–53. doi: 10.1002/bmb.70045 (PMC13206467; doi:10.1002/bmb.70045)
Supplement: Supplementary file 1 — Supporting Information: A. Evaluation sheets. Questionnaire 1: ESM‐Survey: (paper‐based). Questionnaire 2: Pre‐test: (online). Questionnaire 3: Post‐test: (online). [file BMB-54-243-s002.docx]

**Supplementary material A**

**“Bionopoly” as a Gamechanger?**

**Effects of Gamification on learning success, motivation and activation among medical students in a biochemistry course**

Eva Stapfer^1^, Achim Schneider^2^, Ernestine Saumweber^1^ and Susanne J. Kühl^1^

*1 Institute of Biochemistry and Molecular Biology, Medical Faculty, Ulm University, Ulm, Germany*

*2 Medical Faculty, Office of the Dean of Studies, Ulm University, Ulm, Germany*

*Running head:* Effects of gamification on students’ learning

**Corresponding author:**

Prof. Dr. Susanne J. Kühl, Master of Medical Education (MME)

Institute of Biochemistry and Molecular Biology, Ulm University

Albert-Einstein-Allee 11

89081 Ulm, Germany

susanne.kuehl@uni-ulm.de

**Evaluation Sheets**

Only those items that were evaluated and explained in this study are shown. The Pre- and Post-questionnaire was made available online via “unipark” for the students of both groups (control and gaming group). The ESM-survey (experiential sampling method) was handed out in paper form.

**Questionnaire 1: ESM-Survey: (paper-based)**

**Coding**

To ensure an anonymous allocation of data from further surveys, please proceed as follows:

| - Enter the first letter of your place of birth (e.g. U for Ulm): |  | *free text option* |
| --- | --- | --- |
|  |  |  |
| - Enter the first and second letters of your mother's first name (e.g. MO for Monica): |  | *free text option* |
|  |  |  |
| - Enter the first and second letters of your father's first name (e.g. CA for Carl): |  | *free text option* |
|  |  |  |
| - Enter the month of your mother's birth in numbers (two digits, e.g. 06 for June): |  | *free text option* |

**The questions were all answered with a Likert-type scale:**

| **1** | **2** | **3** | **4** | **5** | **6** |
| --- | --- | --- | --- | --- | --- |
| strongly disagree | disagree | rather disagree | rather agree | agree | strongly agree |

- My motivation is high.
- My concentration is high.
- My interest is high.

**Questionnaire 2: Pre-test: (online)**

**Consent**

| I give consent to data processing and data transfer for teaching research purposes by the Medical Faculty of Ulm University. |  | Yes |
| --- | --- | --- |
|  |  | No |

**Coding**

To ensure an anonymous allocation of data from further surveys, please proceed as follows:

| - Enter the first letter of your place of birth (e.g. U for Ulm): |  | *free text option* |
| --- | --- | --- |
|  |  |  |
| - Enter the first and second letters of your mother's first name (e.g. MO for Monica): |  | *free text option* |
|  |  |  |
| - Enter the first and second letters of your father's first name (e.g. CA for Carl): |  | *free text option* |
|  |  |  |
| - Enter the month of your mother's birth in numbers (two digits, e.g. 06 for June): |  | *free text option* |

**Socio-demographic data**

| - Age: | |  | *free text option* |
| --- | --- | --- | --- |
|  | |  |  |
| - Gender: | Female |  |  |
|  | Male |  |  |
|  | Diverse (please specify:) |  | *free text option* |
|  | Not specified |  |  |
|  | |  |  |

**Knowledge acquisition**

| 1. Which of these proteins is NOT fibrillar? | Actin | | |
| --- | --- | --- | --- |
|  | Histones | | |
|  | Myosin | | |
|  | Collagens | | |
|  | Keratin | | |
|  | |  |  |
| 1. Which of these proteins is NOT globular? | Globulin | | |
|  | Globin | | |
|  | Myosin | | |
|  | Histones | | |
|  | Protamine | | |
|  |  | | |
| 1. Which chemical bond is the weakest? | Covalent bond | | |
|  | Ionic bond | | |
|  | Dipole-Dipole interaction | | |
|  | Hydrogen bond | | |
|  | Van-der-Waals-interaction | | |
|  |  | | |
| 1. Which sentence CORRECTLY describes acid precipitation?   Acid precipitation leads to the precipitation of proteins... | by dissolving the disulphide bridges. | | |
|  | by breaking the hydrogen bonds and ionic bonds. | | |
|  | by the formation of chelate complexes. | | |
|  | through the breakage of van der Waals interactions. | | |
|  | by changing the covalent bonds. | | |
|  |  | | |
| 1. Which sentence CORRECTLY describes the precipitation of heavy metals?   The precipitation of heavy metals leads to the precipitation of proteins… | by dissolving the disulphide bridges. | | |
|  | by breaking the hydrogen bonds and ionic bonds. | | |
|  | by changing the covalent bonds. | | |
|  | through the breakage of van der Waals interactions. | | |
|  | by the formation of chelate complexes. | | |
|  |  | | |
| 1. Which bond is used to bind protein A to the column material in affinity chromatography? | Ionic bond | | |
|  | Van-der-Waals-interaction | | |
|  | Hydrophobic interactions | | |
|  | Covalent bond | | |
|  | Hydrogen bond | | |
|  |  | | |
| 1. Which bond is used to bind immunoglobulins to protein A in affinity chromatography? | Ionic bond | | |
|  | Van-der-Waals-interaction | | |
|  | Hydrophobic interactions | | |
|  | Covalent bond | | |
|  | Hydrogen bond | | |
|  |  | | |
| 1. Which statement about serum electrophoresis is INCORRECT? | Serum electrophoresis is a qualitative method. | | |
|  | An agarose gel can be used as the stationary phase. | | |
|  | The mobile phase has a slightly acidic pH (pH<7). | | |
|  | Serum electrophoresis separates proteins on the basis of size, taking into account the total negative charge of the proteins in an alkaline environment. | | |
|  | Coomassie Brilliant Blue stains amino acids non-specifically. | | |
|  |  | | |
| 1. Serum electrophoresis takes place in a buffer, depending on which the proteins migrate to the anode or cathode.   Which answer CORRECTLY completes the following sentence?  In an acidic buffer, proteins are… | negatively charged and move to the cathode. | | |
|  | positively charged and move to the cathode. | | |
|  | positively charged and move to the anode. | | |
|  | negatively charged and move to the anode. | | |
|  | negatively charged and move to the cathode. | | |
|  |  | | |
| 1. What scientific question can be formulated using the principle of a Bradford assay? | How long are the isolated proteins? | | |
|  | What is the respective quantity of the isolated proteins? | | |
|  | What is the colour of the isolated proteins? | | |
|  | Is the function of the isolated proteins preserved? | | |
|  | How does the size of the isolated proteins differ? | | |
|  |  | | |
| 1. What is the best description of the Bradford assay? | Qualitative method in which the length of proteins is measured. | | |
|  | Qualitative method in which the size of proteins is measured. | | |
|  | Quantitative method in which the amount of protein is measured. | | |
|  | Qualitative method in which the amount of proteins is measured. | | |
|  | Quantitative method in which the length of proteins is measured. | | |
|  |  | | |
| 1. Which statement about the dot blot is CORRECT? | The aim of the experiment is to determine the size of the found protein. | | |
|  | To detect the protein, an antibody is added, the antibody then binds to the membrane. | | |
|  | The aim of the experiment is to determine whether the protein is in solution X or Y. | | |
|  | Negative controls are not required for dot blotting. | | |
|  | The antigens added in step 2 enzymatically convert the substrate into a colour. | | |
|  |  | | |
| 1. What is the HA epitope used in the dot blot?   It is... | an antibody. | | |
|  |  the dye. | | |
|  |  the protein solution. | | |
|  |  an enzyme. | | |
|  |  an antigen. | | |
|  |  | | |
| 1. Which statement about the practical experiments is CORRECT? | Serum electrophoresis cannot be done after affinity chromatography. | | |
|  | The Bradford assay is a method for the quantitative analysis of proteins. | | |
|  | The Bradford assay is a qualitative protein assay. | | |
|  | Serum electrophoresis is a method for the quantitative anlysis of proteins. | | |
|  | Affinity chromatography is less specific than salt-induced precipitation of proteins. | | |
|  |  | | |
| 1. Why is the serum passed 3 times over the column during affinity chromatography?   To maximise the probability of binding... | the antigen to the antibody. | | |
|  | protein A to the carrier material. | | |
|  | the immunoglobulin to the antigen. | | |
|  | the immunoglobulin to protein A. | | |
|  | the antibody directly to the carrier material. | | |
|  |  | | |
| 1. Which combination of percentage and protein fraction in serum (from a patient sample in the physiological condition) is CORRECT? | 60% albumin | | |
|  | 2,5% alpha2-globulins | | |
|  | 30% alpha1-globulins | | |
|  | 15% beta-globulins | | |
|  | 25% gamma-globulins | | |
|  |  | | |
| 1. In which of the fractions of serum electrophoresis are acute phase proteins found? | Albumin | | |
|  | Omega-globulins | | |
|  | Alpha2-globulins | | |
|  | Beta-globulins | | |
|  | Gamma-globulins | | |
|  |  | | |
| 1. Which combination of serum protein and the corresponding protein is CORRECT? | Plasminogen and gamma-globulins | | |
|  | IgM and alpha-Globulins | | |
|  | Prothrombin and alpha-globulins | | |
|  | Plasminogen and beta-globulins | | |
|  | Transferrin and gamma-globulins | | |
|  |  | | |
| 1. The blood serum of a healthy medical student is analysed.   Which combination of serum proteins and the corresponding percentage is INCORRECT? | 60% albumin | | |
|  | 15% alpha1-globulins | | |
|  | 7,5% alpha2-globulins | | |
|  | 17% gamma-globulins | | |
|  | 12% beta-globulins | | |

**Questionnaire 3: Post-test: (online)**

**Knowledge acquisition**

The questions are identical to the knowledge acquisition in questionnaire 2.

**Evaluation**

**Impact factors on learning:**

|  |  |  | *positive impact* | *no impact* | *negative impact* |
| --- | --- | --- | --- | --- | --- |
| How did the following factors affect your learning in course 9? | interactive teaching |  |  |  |  |
|  | group dynamics |  |  |  |  |
|  | presentation slides |  |  |  |  |
|  | teacher’s instructional style |  |  |  |  |
|  | game „Bionopoly“ |  |  |  |  |
|  | others: |  | *free text option* | | |

**General satisfaction**

|  | *school grades (6 is the best grade)* |
| --- | --- |
| I rate course 9 with the following school grade: | 1 |
|  | 2 |
|  | 3 |
|  | 4 |
|  | 5 |
|  | 6 |
| In this free text field, you have the opportunity to praise, criticise or make specific suggestions for improvement: | *free text option* |

**The following questions were answered with a Likert-type scale**:

| **1** | **2** | **3** | **4** | **5** | **6** |
| --- | --- | --- | --- | --- | --- |
| strongly disagree | disagree | rather disagree | rather agree | agree | strongly agree |

**ARCS-Model (Attention, Relevance, Confidence and Satisfaction) for motivation:**

- **Attention**: The way the learning materials are presented helps me to focus my attention.
- **Relevance:** After participating in the learning activity, I was motivated to learn more about proteins.
- **Confidence:** I’m confident that I will be able to accomplish all the activities.
- **Satisfaction:** I am satisfied with my learning success in today’s learning activities.

**Specific Question about the game “Bionopoly” (ONLY for gaming group):**

- Playing the game „Bionopoly“ in course 9 helped me to better understand the topic proteins.

**Motivational Factors:**

| What motivated you to actively participate in course 9?  (multiple choice) | fun |  |  |
| --- | --- | --- | --- |
|  | intrinsic motivation |  |  |
|  | competition |  |  |
|  | group dynamics |  |  |
|  | none of the above |  |  |
|  | others: |  | *free text option* |
