# Supplementary material for: “Bionopoly” as a Gamechanger? Effects of Gamification on Learning Success, Motivation and Activation Among Medical Students in a Biochemistry Course
Source: Biochem Mol Biol Educ. 2026 Mar 6;54(3):243–53. doi: 10.1002/bmb.70045 (PMC13206467; doi:10.1002/bmb.70045)
Supplement: Supplementary file 2 — Supporting Information: B. Comments—General satisfaction. In the control group, 24 of the participants (N = 74) used the comment function, which corresponds to 32%, compared to nine participants (N = 60) in the gaming group who commented, which corresponds to 15%. The texts were sorted according to the content of the improvement suggestion and according to praise. A text can be categorized both as a suggestion for improvement and as praise. If a comment addressed several of the topics, the comment was assigned to all of these topics and categorized under NK (for the number of comments on this topic). The number N in the table stands for the total number of comments made in this group. According to the system above, N does not correspond to the sum of NK. [file BMB-54-243-s001.docx]

**Supplementary material B**

**“Bionopoly” as a Gamechanger?**

**Effects of Gamification on learning success, motivation and activation among medical students in a biochemistry course**

Eva Stapfer^1^, Achim Schneider^2^, Ernestine Saumweber^1^ and Susanne J. Kühl^1^

*1 Institute of Biochemistry and Molecular Biology, Medical Faculty, Ulm University, Ulm, Germany*

*2 Medical Faculty, Office of the Dean of Studies, Ulm University, Ulm, Germany*

*Running head:* Effects of gamification on students’ learning

**Corresponding author:**

Prof. Dr. Susanne J. Kühl, Master of Medical Education (MME)

Institute of Biochemistry and Molecular Biology, Ulm University

Albert-Einstein-Allee 11

89081 Ulm, Germany

susanne.kuehl@uni-ulm.de

**Comments – General satisfaction:**

In the control group, 24 of the participants (N=74) used the comment function, which corresponds to 32%, compared to 9 participants (N=60) in the gaming group who commented, which corresponds to 15%. The texts were sorted according to the content of the improvement suggestion and according to praise. A text can be categorised both as a suggestion for improvement and as praise. If a comment addressed several of the topics, the comment was assigned to all of these topics and categorised under N_K_ (for the number of comments on this topic). The number N in the table stands for the total number of comments made in this group. According to the system above, N does not correspond to the sum of N_K_.

| **Topic** | **N_K_** | **Control group (N = 24)** | **N_K_** | **Gaming group (N = 9)** |
| --- | --- | --- | --- | --- |
| **Duration/ Time (Criticism)** | 9 | „Simply too long.“  „It takes a long time, but […]“  “Please Arrange the order of the experiments so that you don't have an hour between the practical course and the seminar just to wait for the result [...].”  “The waiting times for the experiments are clearly too long.”  “Simply too long in terms of duration to maintain concentration.”  “[...] Experiments take a very long time”  "It would be better with more quality instead of quantity. [...] but it doesn't make sense."  “Very long and you wait 60 minutes.”  “Too long, please reduce waiting times.”  “Waiting too long, especially waiting for electrophoresis, is very unnecessary.” | 5 | “Waiting 1.5 hours for a bit of blue is just not that cool”  "[...] neither the strength nor the concentration, and after such an unnecessarily long internship, no desire to sit in the evening seminar. [...]“  ”The waiting time for electrophoresis was too long." “Long idle time until gel electrophoresis [...]!”  “Not such long waiting times!” |
| **Structure of the course**  **(Criticism)** | 6 | "The group had to wait 1.5 hours [...], [...]. It would be much better to have the seminar in these 1.5 hours, because then you could concentrate much better."  "[...] you should do the evening seminar during the electrophoresis period. [...] Alternatively, omit the experiment and show what it should look like."  “You could move the evening seminar to the time when gel electrophoresis is taking place.”  “Debriefing between the end of the lab and the gel electrophoresis.”  “During the hour in which you wait for the evaluation of the experiment, you could already do the evening seminar.”  “I am in favor of block teaching for the internships before the exam and less spread out over months.” | 2 | “The 0.5h break while the color binds at the end could be better bridged by the seminar instead of just waiting.”  “Perhaps in future you could use the results from previous courses for the electrophoresis so that you don't have to wait an hour.” |
| **Repetition of content**  **(Criticism)** | 2 | “Avoid duplication with online videos and seminars.”  “Too often the same content.” | 0 | --- |
| **Concentration**  **(Criticism)** | 5 | “However, the fun and concentration wanes in the 10th hour of university [...]”  “Nobody can concentrate that long.”  “Well-meant, but you're just not receptive for that long.”  “Simply too long in terms of duration to maintain concentration.”  “[...] concentration wanes over time, maybe you could try to make everything a bit more varied.” | 1 | "[...] neither the strength nor the concentration, and after such an unnecessarily long course, no desire to sit in on the evening seminar. [...]" |
| **Praise** | 4 | “ [...], but the time passes quickly due to the permanent occupation and it is very interesting.”  “Preseminar very good, [...]”  “ [...] The lecturer is lovely, [...].”  "I really like the slides! I find them very instructive." | 3 | “Didactically amazing [...].”  “Was great fun.”  “[...] Bionopoly was a lot of fun.” |
